# Acute Hepatitis Induced by Lyprinol, the Lipid Extract of the Green-Lipped Mussel (*Perna canaliculus*), in a Patient with Polyarthrosis

**DOI:** 10.1155/2012/135146

**Published:** 2012-05-31

**Authors:** Amr Abdulazim, Marion Hädrich, Matteo Montani, Nasser Semmo

**Affiliations:** ^1^University Clinic for Visceral Surgery and Medicine, INSELSPITAL, Freiburgstraße 18, 3010 Bern, Switzerland; ^2^Institute of Pathology, University of Bern, 3010 Bern, Switzerland

## Abstract

Lyprinol, the lipid extract of the green-lipped mussel (*Perna canaliculus*), is a readily and freely available agent with a putative anti-inflammatory impact. It has already found application as a complementary and supplementary treatment of osteoarthritis, rheumatoid arthritis, asthma, and cancer. So far no major side effects for Lyprinol have been reported, yet. Here, we present the case of a 76-year-old woman with acutely exacerbating abdominal pain and highly elevated liver transaminases while taking Lyprinol as a complementary treatment of polyarthrosis.

## 1. Case Report

A 76-year-old woman with a six-month history of recurrent, nocturnal, right epigastric pain radiating to the right shoulder and to the back presented to her general practitioner with an acute exacerbation of abdominal pain. An outpatient blood analysis revealed elevated liver transaminases suspicious of an acute hepatitis; thus the patient was referred to our department. On admission the patient had a C-reactive protein of 4 mg/L (normal <5 mg/L), total bilirubin 39 *μ*mol/L (<17 *μ*mol/L), aspartate aminotransferase (AST) 1886 IU/L (<35 IU/L), alanine aminotransferase (ALT) 1462 IU/L (<35 IU/L), *γ*-glutamyltransferase (*γ*-GT) 468 IU/L (5–36 IU/L), alkaline phosphatase (ALP) 174 IU/L (35–105 IU/L), and lipase 38 IU/L (13–60 IU/L). Physical examination did not reveal any abnormalities except for a slight right abdominal tenderness on palpation and bilateral Heberden nodes. Ultrasonography showed several concrements of the gall bladder, with a maximum diameter of 1.4 cm. An abdominal computerized tomography (CT) scan showed neither intra- or extrahepatic cholestasis nor any other pathologies of the abdomen including the liver. Hepatitis serology was significant for anti-hepatitis A virus IgG positivity, indicative of a status after hepatitis A infection, whereas hepatitis B and C infections could be ruled out.

**Figure 1:**
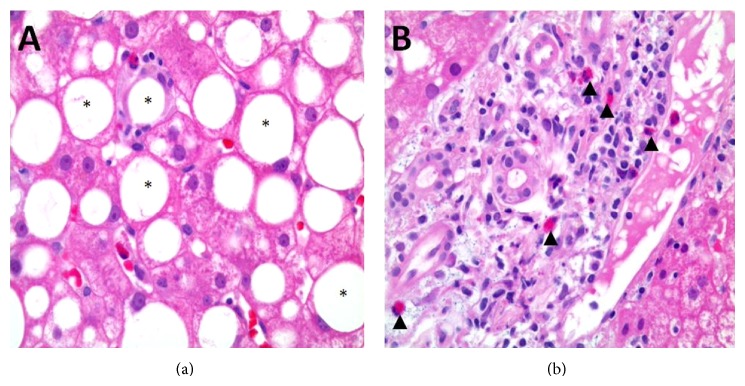
(a) and (b) Representative specimen of the liver biopsy showing a mixture of a rampant steatosis (∗), a lipogranuloma as a hint to florid steatohepatitis (a) as well as minimal portal field edema, and portal eosinophilic infiltration (▲) suggestive of a medical or toxic injury (b).

There was no evidence for an autoimmune hepatitis. The patient was on pantoprazole 20 mg/d, vitamin D_3_ 100 *μ*g/d, and rose-hip powder long time before complaints and admission. Additionally, she started taking the lipid extract of the green-lipped mussel (Lyprinol) 2 months prior to admission as a complementary treatment of polyarthrosis, raising the suspicion of a drug-induced acute hepatitis. Strikingly, liver biopsy (Figure 1) was compatible with a drug-induced hepatitis. Indeed, after withdrawal of Lyprinol transaminases significantly decreased by a factor of 3–9, and the patient could be discharged after few days. At an outpatient control six weeks after hospitalisation, the patient had no complaints and blood values were completely normalized.

## 2. Discussion

Lyprinol, the lipid extract of the green-lipped mussel (*Perna canaliculus*) endemic to New Zealand, was launched to the world market in 1998 as a readily and freely available anti-inflammatory agent [1]. Ever since, Lyprinol has been subject to a number of clinical trials to study its effects on osteoarthritis, rheumatoid arthritis, asthma, and cancer [1–4]. Lyprinol contains the *ω*-3 polyunsaturated fatty acids eicosapentaenoic acid, docosahexaenoic acid, and 7,11,14,17-eicosatetraenoic acid, which are structurally similar to arachidonic acid, the precursor to the inflammatory agents prostaglandins and leukotrienes [5]. Lyprinol presumably inhibits the cyclooxygenase enzyme and the lipoxygenase enzyme competitively, reducing prostaglandin and leukotriene levels, thus exerting its putative anti-inflammatory function [4]. It has further been reported to decrease the ability of lipopolysaccharide to stimulate tumor necrosis factor-*α* (TNF-*α*) and interferon-*γ* (IFN-*γ*) and to inhibit the production of interleukin-1, interleukin-2, interleukin-6, and IgG [5].

So far Lyprinol is assumed to be a safe agent with virtually no major complications. Solely two cases of hepatic dysfunction are reported in the literature [1]. However, in both patients liver values were already elevated prior to Lyprinol, and peak values after Lyprinol administration in our patient were sixfold of those presented earlier [1]. Additionally, one of the patients described had a disease with significant hepatic involvement most likely being the major reason for liver value elevation, whereas our patient had no underlying hepatic disease or involvement.

As Lyprinol is available without prescription and its merits are highly praised, it may possibly be considered by many patients as a supplementary agent. This results in a high prevalence of patients taking Lyprinol, thus increasing the incidence of potential Lyprinol-associated side effects. This illustrated unusual case demonstrates that general practitioners as well as clinical doctors should take severe acute hepatitis into consideration as a drug-induced side effect in patients presenting with acute abdominal pain, elevated liver transaminases, and a history of Lyprinol intake.
